# Dietary fat intake and liver cancer risk: A prospective cohort study in Chinese women

**DOI:** 10.20892/j.issn.2095-3941.2020.0633

**Published:** 2021-09-21

**Authors:** Xiaowei Ji, Jing Wang, Zhuoying Li, Qiuming Shen, Jiayi Tuo, Jinghao Bi, Yuting Tan, Honglan Li, Yongbing Xiang

**Affiliations:** 1 State Key Laboratory of Oncogene and Related Genes & Department of Epidemiology, Shanghai Cancer Institute, Renji Hospital, Shanghai Jiao Tong University School of Medicine, Shanghai 200032, China; 2 School of Public Health, Shanghai Jiao Tong University School of Medicine, Shanghai 200025, China

**Keywords:** Liver cancer, dietary fat, prospective cohort, Chinese women

## Abstract

**Objective:**

This study aimed to determine whether dietary fat intake increased liver cancer risk in Chinese women from a prospective population-based cohort.

**Methods:**

A total of 72,704 Chinese women were followed up from the time of baseline recruitment (1996–2000) to the end of 2016. Dietary fat intake was calculated using a validated food frequency questionnaire. The Cox regression model was used to assess the hazard ratio (HR) and 95% confidence intervals (CI) for dietary fat intake and liver cancer risk.

**Results:**

We identified 252 incident liver cancer cases out of 1,267,845 person-years during the overall follow-up time. Null associations, neither in quartiles nor per standard deviation (SD) increment, were detected between liver cancer risk and dietary total fat, fat subtypes and subtype ratios, and food sources. The HR (95% CI) of the 1-SD increment was 1.03 (0.90–1.17) for total fat, 1.06 (0.93–1.20) for saturated fat, 1.06 (0.93–1.21) for monounsaturated fat, and 1.00 (0.89–1.13) for polyunsaturated fat. Similar null associations were observed in stratification analyses according to body mass index and menopausal status.

**Conclusions:**

In our prospective cohort study, no significant association was observed in Chinese women between dietary fat and liver cancer risk, and in stratification and sensitivity analyses.

## Introduction

Liver cancer is the ninth most common cancer in women worldwide, with 273,357 new cases in 2020^1^, but 244,506 incident cases in 2018^2^. A statistical analysis of incidence rates in China reported that liver cancer represented the seventh most common cancer in women, with 96,000 cases in 2015^3^. Hepatocellular carcinoma (HCC), the major histological type of primary liver cancer, accounted for 75%–85% of total liver cancer cases^4^. Chronic hepatitis B and C virus (HBV and HCV) infections are the most frequent liver cancer causes, comprising 80% of all HCC cases globally^5^. Liver cancer also shows a sex-specific disparity in terms of incidence between male and female patients; males are 3 times more likely to develop HCC than females^6^. Hormones are suggested to be the most important cause for sex discrepancy in HCC carcinogenesis. Manieri et al.^7^ and Greten^8^ suggested reduced adiponectin levels were responsible for the increased incidence of HCC in men. Another study suggested that serum interleukin-6 (IL-6) levels can play a critical role in males^9^. IL-6 has been reported to be associated with adiponectin expression, and adiponectin levels may explain the sex-specific disparity in HCC carcinogenesis.

Growing attention has been recently addressed towards the role of nutritional factors in the pathogenesis of common cancers. One explanation is that quantity and quality of nutrients and foods have deep impacts on the pro-inflammatory carcinogenic effects or anti-cancer immune responses^10^. In recent years, new epidemiological evidence based on 2 large prospective cohorts conducted in the U.S. involving the Nurses’ Health Study (NHS) and the Health Professionals Follow-up Study (HPFS) has supported a change in research on liver cancer risk factors. By considering dietary patterns, these 2 cohort studies have shown that better adherence to the AHEI-2010 diet pattern might decrease the risk of developing HCC^11^. In terms of food or food groups, increased intakes of whole grains, cereal fiber, and bran were associated with a reduced risk of HCC^12^. In contrast, processed red meat intake might be associated with a higher risk of HCC, while poultry or fish intake might be associated with a lower risk of HCC^13^. Regarding specific nutrients, higher intakes of vegetable fat and polyunsaturated fat (PUFA) were found to be associated with lower risks of HCC^14^. These studies both confirmed that a plant-based low carbohydrate diet and dietary restriction of carbohydrates from refined grains were associated with a lower risk of HCC. In conclusion, exchanging carbohydrates for more plant-based fats and proteins, substituting plant fat and protein for carbohydrates, particularly refined grains, might decrease the risk of HCC^15^. The above mentioned evidence highlighted the importance of diet nutrients on liver cancer incidence and has stimulated studies of alimentary control and liver cancer risks. The NHS and HPFS also suggested that greater adherence to the type 2 diabetes mellitus (T2DM) diet was associated with a decreased risk of developing HCC among U.S. men and women^16^.

Only 3 case-control and 4 cohort studies have been performed to determine the associations between dietary fat and liver cancer risk. These studies were conducted in the U.S., Europe, and Singapore (for Chinese residents), but further details of these associations had not been determined in mainland China. According to the differences in dietary cultures between China and other countries, and variations of eating habits for different sexes, it is worth characterizing the associations between dietary fat and liver cancer risk in Chinese women. Epidemiological evidence comparing diet and cancer incidence risk in women has been reported for breast cancer^17,18^, endometrial cancer^19^, epithelial ovarian cancer^20^, and colorectal cancer^21^, but not for liver cancer. In this population-based prospective cohort study, we determined whether dietary intakes of total fat, fat subtypes, and subtype ratios and food sources increased liver cancer in Chinese women. We also aimed to clarify the effects of dietary fatty acids on liver carcinogenesis in women.

## Materials and methods

### Study population

The Shanghai Women’s Health Study (SWHS) is a population-based prospective cohort study, which enrolled 74,940 women between 40 and 70 years of age. The study was conducted in urban Shanghai, and the baseline surveys were conducted from 1996 to 2000^22^. Participants in the SWHS were interviewed by trained interviewers to complete the baseline surveys, including demographic information, occupation, dietary habits, physical activity, smoking, alcohol consumption, and tea consumption, and the personal medical history of chronic diseases and family history of cancer were also recorded. Informed consent was obtained from each recruited individual. In this study, participants who met the following criteria were excluded: 1) diagnosis of cancer *in situ* during follow-up (*n* = 135), 2) cancer death with no cancer type or diagnosis date (*n* = 244), 3) cancer at baseline (*n* = 1,598), 4) loss to follow-up shortly after enrollment (*n* = 3), 5) a cancer diagnosis that could not be confirmed (*n* = 67), 6) extreme values for total calorie intake (< 500 or > 3,500 kcal/day) (*n* = 121), and 7) participants with missing data for any covariates of interest were also excluded (*n* = 68). After these exclusions, a total of 72,704 participants were finally included in the study. This study was approved by the Renji Hospital Ethics Committee of Shanghai Jiao Tong University School of Medicine (KY2019-197).

### Measurement of dietary intakes

A previously validated semi-quantitative food frequency questionnaire (FFQ) was adopted to evaluate dietary food intake within this cohort and was administered by an in-person interview^23^. A total of 77 food items and food groups were included in the FFQ, which covered approximately 90% of commonly consumed foods in urban Shanghai in 1996. All study participants were interviewed twice to provide names and amounts of food they consumed over the past 24 h. The 24 h dietary recall interview was chosen to obtain detailed information about all food and beverages consumed by each participant in the past 24 h. An unannounced in-person interview was conducted to assess the frequency (categorized by daily, weekly, monthly, yearly, or never) and the quantity (amounted with Liangs, 1 Liang = 50 g) of food consumption per time. Daily nutrient intakes were calculated from the FFQs using the nutrient content of each food based on the 2002 Chinese Food Composition Table^24^.

Total fat was calculated from all food items included in the FFQ, except for cooking oils. Due to some limitations, only 4 leading food sources of dietary consumption were presented including soy, vegetable, fruit, and red meat. Because of the low content of dietary fat from vegetables, fruit, and soy, we summed them and presented them as “plant fat”. “Red meat fat” was set as a single group, according to the relatively higher consumption of fat in red meat. Finally, the remaining source of dietary fat was defined as “other fat”. Three food sources of dietary fat were therefore formally presented. Three fat subtypes including saturated fat, monounsaturated fat (MUFA), and polyunsaturated fat (PUFA) were classified. Continuous scales of total fat, fat subtypes, and food sources were graded into quartiles and created with dummy variables. Fat composition ratios including MUFA to saturated fat (M:S), PUFA to saturated fat (P:S), and MUFA and PUFA to saturated fat [(M + P):S] ratios were calculated to determine their associations with liver cancer incidence. We also determined the associations between dietary fat (including cooking oils) and liver cancer risk. However, because the added fat (oils) data were not as accurate as the dietary fat data as comprehensively discussed in the “limitation” section of this manuscript, these results are provided as a supplementary file.

### Follow-up and case confirmation

All cohort members were followed up for cancer occurrence through in-person surveys every 3–4 years. The research outcomes were annually linked with the databases of the Shanghai Cancer Registry, the Shanghai Vital Statistics Registry, and the Shanghai Resident Registry. In total, 5 follow-up surveys on outcomes were performed with response rates of 99.7% (2000–2002), 98.7% (2002–2004), 94.9% (2004–2006), 92.3% (2007–2010), and 91.1% (2012–2017). Person-year (PY) estimation for all cohort members started at baseline (i.e., the incidence of liver cancer) or when a right-censoring event occurred (i.e., death, loss to follow-up, or December 31, 2016), whichever came first. All liver cancer diagnoses were verified through home visits and rechecked with the medical reports from hospitals. The medical charts were reviewed by clinical and pathology experts. Cancers were coded according to the International Classification of Disease, Ninth Revision (ICD-9). Liver cancer was defined as a primary malignant tumor with an ICD-9 code of 155 defined as “malignant neoplasm of liver and intrahepatic bile ducts”^25^.

### Statistical analysis

Means and standard deviations (SDs) were calculated for continuous variables, while counts and proportions were presented for categorical variables. The Cox proportional hazard regression model was used to evaluate the association between dietary fat intake and liver cancer incidence by using hazard ratio (HR) and 95% confidence interval (CI). Follow-up time was determined as the underlying time metric. The Schoenfeld residual method was used to check the proportional hazard assumptions for all dietary fat items, but no violations were detected.

HRs (95% CIs) of both quartile categories and the 1-SD increment of total fat, fat subtypes and subtype ratios, and fat food sources were estimated in the main analyses. The nutrient residual model was used to calculate energy-adjusted total fat and specific fats^26^. Two Cox regression models were involved in this study. The first model was adjusted by age (continuous) and total calorie intake (kcal/day, quartile). The second model was a multivariate model, with the confounding variables including: age (continuous), body mass index (BMI, calculated as weight/height^2^, categorized into 4 groups as < 18.5, ~ 23.9, ~ 27.9, and ≥ 28 kg/m^2^, according to the Chinese Joint Committee for Developing Chinese Guidelines and the Working Group on Obesity in China)^27^, education (4 categories: elementary school and below, middle school, high school, college and above), annual family income (yuan, 4 categories: < 10,000, ~ 19,999, ~ 29,999, ≥ 30,000), occupation (4 categories: professional, housewife, clerical, and manual workers), menopausal status (yes/no), smoking (defined as “ever smoked at least 1 cigarette/day for more than 6 months,” (yes/no), alcohol consumption (defined as “ever drank alcohol at least 3 times/week for more than 6 months,” yes/no), tea drinking (defined as “ever drank tea at least 3 times/week for more than 6 months,” yes/no), physical activity [metabolic equivalent (MET)-h/week, quartile], total calorie intake (kcal/day, quartile), personal medical history of T2DM (yes/no), cholelithiasis (yes/no), chronic hepatitis (yes/no), and family history of liver cancer (yes/no). Trend tests were conducted across the energy-adjusted quartiles of dietary fats.

Prior exploratory subgroup analyses were conducted to evaluate the associations between total fat, fat subtypes and subtype ratios, and food sources and liver cancer in participants who had different characteristics such as BMI (< 24 *vs.* ≥ 24 kg/m^2^) and menopausal status (yes *vs.* no). The interactive effects of these factors were also determined. As previous evidence has indicated that smoking and alcohol consumption were associated with liver cancer development^28^, we should have conducted subgroup analyses of these parameters. However, due to the limited proportion of smokers and alcohol consumers in our SWHS (2007/72,704 and 1631/72,704), these analyses were not conducted. Three sensitivity analyses were conducted as follows: 1) to eliminate bias due to possible reverse causation in cohort studies, we excluded participants whose follow-up time was less than 2 years; 2) we excluded participants who had T2DM at baseline; 3) the nutrient density method^26^ was adopted to re-analyze the associations between all dietary fats and liver cancer risks.

All analyses were conducted using SAS version 9.4 (SAS Institute, Cary, NC, USA). All *P*-values were two-sided, and *P* < 0.05 was considered statistically significant.

## Results

A total of 252 new cases of female liver cancer were identified during over 1.26 million person-years of the follow-up period, from baseline to the end of 2016, with an average follow-up time of 17.44 years for each person. The cumulative incidence density and rate of liver cancer were 0.20/1,000 person-years and 3.47/1,000 participants during the follow-up time, respectively. Participants who were older and menopausal, and had a higher BMI, medical history of hepatitis, cholelithiasis, and T2DM, and family history of liver cancer were more likely to develop liver cancer. A decreased probability to develop liver cancer was detected in women who had consumed tea. Imbalanced discrepancies of education background, occupation, and family income were presented in non-cases and liver cancer cases. After categorizing participants according to the quartiles of dietary fat intake, a statistical significance was observed in all factors, except for family history of liver cancer (**Table 1**).

**Table 1:** Baseline demographic and lifestyle characteristics of liver cancer cases and non-cases (SWHS, 1996–2016)

	non-cases (*n* = 72,452)	liver cancer cases (*n* = 252)	*P*	Dietary total fat in all cohort members				*P*
				Q1 (*n* = 18,176)	Q2 (*n* = 18,176)	Q3 (*n* = 18,176)	Q4 (*n* = 18,176)	
Age	52.48 ± 9.05	58.48 ± 8.27	< 0.0001	54.47 ± 9.12	52.57 ± 9.09	51.87 ± 8.97	51.09 ± 8.68	< 0.0001
BMI (kg/m^2^)	24 (3.42)	25.01 (3.77)	< 0.0001	24.79 (3.6)	24 (3.4)	23.69 (3.33)	23.55 (3.21)	< 0.0001
Physical activity (MET-h/week)	106.57 (45.09)	106.36 (48.3)	0.942	111.65 (46.5)	106.82 (44.78)	104.33 (43.99)	103.46 (44.63)	< 0.0001
Calorie intake (kcal/day)	1673.89 (393.73)	1627.46 (391.02)	0.062	1769.98 (390.07)	1595.64 (352.98)	1600.84 (362.87)	1728.45 (433.6)	< 0.0001
Education			< 0.0001					< 0.0001
Elementary school and below	15,393 (21.25)	107 (42.46)		6,202 (34.12)	3,946 (21.71)	3,035 (16.7)	2,317 (12.75)	
Middle school	27,003 (37.27)	62 (24.6)		6,805 (37.44)	6,900 (37.96)	6,778 (37.29)	6,582 (36.21)	
High school	20,262 (27.97)	59 (23.41)		3,722 (20.48)	5,073 (27.91)	5,516 (30.35)	6,010 (33.07)	
College and above	9,794 (13.52)	24 (9.52)		1,447 (7.96)	2,257 (12.42)	2,847 (15.66)	3,267 (17.97)	
Occupation			0.034					< 0.0001
Professional	20,618 (28.46)	57 (22.62)		3,628 (19.96)	5,010 (27.56)	5,803 (31.93)	6,234 (34.3)	
Housewife	265 (0.37)	3 (1.19)		96 (0.53)	73 (0.4)	44 (0.24)	55 (0.3)	
Clerical	15,024 (20.74)	58 (23.02)		3,783 (20.81)	3,744 (20.6)	3,759 (20.68)	3,796 (20.88)	
Manual workers	36,545 (50.44)	134 (53.17)		10,669 (58.7)	9,349 (51.44)	8,570 (47.15)	8,091 (44.51)	
Family income (yuan/year)			0.001					< 0.0001
< 10,000	11,632 (16.05)	59 (23.41)		3,657 (20.12)	2,997 (16.49)	2,604 (14.33)	2,433 (13.39)	
10,000-	27,693 (38.22)	93 (36.9)		7,559 (41.59)	7,054 (38.81)	6,683 (36.77)	6,490 (35.71)	
20,000-	20,373 (28.12)	74 (29.37)		4,600 (25.31)	5,100 (28.06)	5,352 (29.45)	5,395 (29.68)	
30,000	12,754 (17.6)	26 (10.32)		2,360 (12.98)	3,025 (16.64)	3,537 (19.46)	3,858 (21.23)	
Smoking status (yes)	1,996 (2.75)	11 (4.37)	0.119	623 (3.43)	458 (2.52)	440 (2.42)	486 (2.67)	< 0.0001
Alcohol drinking status (yes)	1,627 (2.25)	4 (1.59)	0.481	329 (1.81)	337 (1.85)	399 (2.2)	566 (3.11)	< 0.0001
Tea drinking status (yes)	21,689 (29.94)	54 (21.43)	0.003	4,170 (22.94)	5,285 (29.08)	5,808 (31.95)	6,480 (35.65)	< 0.0001
Menopausal status (yes)	35,317 (48.75)	194 (76.98)	< 0.0001	10,596 (58.3)	8,987 (49.44)	8,324 (45.8)	7,604 (41.84)	< 0.0001
Family history of liver cancer	2,360 (3.26)	25 (9.92)	< 0.0001	598 (3.29)	558 (3.07)	641 (3.53)	588 (3.24)	0.105
History of hepatitis	1,830 (2.53)	36 (14.29)	< 0.0001	405 (2.23)	491 (2.7)	485 (2.67)	485 (2.67)	0.011
History of cholelithiasis	8,044 (11.1)	50 (19.84)	< 0.0001	2,506 (13.79)	2,081 (11.45)	1,852 (10.19)	1,655 (9.11)	< 0.0001
History of diabetes	3,099 (4.28)	25 (9.92)	< 0.0001	486 (2.67)	666 (3.66)	826 (4.54)	1,146 (6.31)	< 0.0001

SWHS, Shanghai Women’s Health Study; BMI, body mass index. Values are the means (SD) for continuous variables and count (proportion) for categorical items.

Null associations were found in dietary intakes of total fat, specific fat subtypes, food sources, and liver cancer risks in both regression models. The highest *vs.* lowest quartile of HR (95% CI) was 1.11 (0.79–1.57) for total fat, 1.25 (0.87–1.79) for saturated fat, 1.08 (0.75–1.55) for MUFA, and 1.02 (0.73–1.45) for PUFA after adjusting for multiple confounders, and the corresponding *P*-trend values were 0.611, 0.246, 0.788, and 0.816, respectively. Taking into consideration the M:S, P:S, and (M + P):S ratios, no significance was observed in both models; the highest *vs.* lowest quartiles of HR (95% CI) were 0.88 (0.62–1.25) for the M:S ratio, 0.94 (0.66–1.34) for P:S ratio, and 1.15 (0.80–1.66) for the (M + P):S ratio after adjusting by multivariate. Furthermore, null associations were detected in relation to the food sources of dietary fat. The highest *vs.* lowest quartile of HR (95% CI) were 1.04 (0.73–1.50) for plant fat, 0.83 (0.58–1.19) for red meat fat, and 1.16 (0.80–1.69) for other fat with *P*-trend values of 0.480, 0.615, and 0.442, respectively (**Table 2**). Null associations were detected in relation to cooking oil (**Supplementary Table S1**).

**Table 2:** Adjusted HRs and 95% CIs of liver cancer by quartiles of energy-adjusted^†^ intakes of dietary fats (SWHS, 1996–2016)

	HR (95% CI)				*P_trend_*	HR (95% CI) for 1-SD increment
	Q1	Q2	Q3	Q4		
**Total fat**						
Median	19.1	25.49	30.4	37.91		
Cases/PYs	78/314,140	57/317,582	53/318,083	64/318,040		
Model 1	1.00 (ref.)	0.78 (0.55, 1.10)	0.77 (0.54, 1.10)	1.03 (0.74, 1.44)	0.979	1.00 (0.88, 1.13)
Model 2	1.00 (ref.)	0.81 (0.57, 1.15)	0.83 (0.58, 1.20)	1.11 (0.79, 1.57)	0.611	1.03 (0.90, 1.17)
**Saturated fat**						
Median	4.99	7.17	8.98	11.68		
Cases/PYs	70/314,024	63/316,819	57/318,343	62/318,659		
Model 1	1.00 (ref.)	0.97 (0.69, 1.37)	0.96 (0.67, 1.36)	1.12 (0.80, 1.59)	0.569	1.01 (0.89, 1.15)
Model 2	1.00 (ref.)	1.04 (0.73, 1.47)	1.05 (0.73, 1.51)	1.25 (0.87, 1.79)	0.246	1.06 (0.93, 1.20)
**Monounsaturated fat**						
Median	7.56	10.76	13.31	17.47		
Cases/PYs	73/314,453	66/316,713	56/318,161	57/318,517		
Model 1	1.00 (ref.)	0.96 (0.68, 1.34)	0.87 (0.61, 1.24)	1.00 (0.71, 1.43)	0.850	1.03 (0.91, 1.17)
Model 2	1.00 (ref.)	0.99 (0.70, 1.39)	0.93 (0.65, 1.34)	1.08 (0.75, 1.55)	0.788	1.06 (0.93, 1.21)
**Polyunsaturated fat**						
Median	4.88	6.51	7.88	10.14		
Cases/PYs	69/317,698	55/317,881	59/317,449	69/314,818		
Model 1	1.00 (ref.)	0.80 (0.56, 1.15)	0.86 (0.60, 1.22)	1.00 (0.72, 1.40)	0.900	1.00 (0.89, 1.14)
Model 2	1.00 (ref.)	0.83 (0.58, 1.20)	0.88 (0.62, 1.26)	1.02 (0.73, 1.45)	0.816	1.00 (0.89, 1.13)
**M:S ratio**						
Median	1.24	1.43	1.57	1.8		
Cases/PYs	66/318,339	59/316,417	67/315,637	60/317,452		
Model 1	1.00 (ref.)	0.87 (0.61, 1.24)	0.94 (0.66, 1.33)	0.92 (0.65, 1.30)	0.730	1.00 (0.91, 1.10)
Model 2	1.00 (ref.)	0.83 (0.58, 1.19)	0.90 (0.63, 1.28)	0.88 (0.62, 1.25)	0.591	1.00 (0.90, 1.10)
**P:S ratio**						
Median	0.63	0.81	0.99	1.32		
Cases/PYs	57/320,731	46/318,888	72/315,453	77/312,772		
Model 1	1.00 (ref.)	0.73 (0.50, 1.09)	1.03 (0.72, 1.46)	1.04 (0.73, 1.47)	0.411	1.00 (0.89, 1.11)
Model 2	1.00 (ref.)	0.73 (0.49, 1.08)	0.98 (0.68, 1.40)	0.94 (0.66, 1.34)	0.813	0.99 (0.88, 1.12)
**(M + P):S ratio**						
Median	1.96	2.29	2.58	3.02		
Cases/PYs	50/319,910	63/318,170	65/316,026	74/313,739		
Model 1	1.00 (ref.)	1.22 (0.84, 1.77)	1.13 (0.77, 1.64)	1.26 (0.88, 1.80)	0.308	1.00 (0.90, 1.10)
Model 2	1.00 (ref.)	1.18 (0.81, 1.72)	1.07 (0.73, 1.57)	1.15 (0.80, 1.66)	0.607	0.99 (0.9, 1.10)
**Food sources**						
**Plant fat**						
Median	2.68	4.44	6.13	9.2		
Cases/PYs	55/320,537	53/317,774	74/315,669	70/313,865		
Model 1	1.00 (ref.)	0.90 (0.61, 1.31)	1.20 (0.84, 1.70)	1.09 (0.77, 1.56)	0.326	1.00 (0.88, 1.13)
Model 2	1.00 (ref.)	0.89 (0.61, 1.31)	1.18 (0.83, 1.69)	1.04 (0.73, 1.50)	0.480	0.97 (0.86, 1.10)
**Red meat fat**						
Median	2.84	6.45	9.49	15.23		
Cases/PYs	73/316,187	56/316,055	70/316,976	53/318,627		
Model 1	1.00 (ref.)	0.72 (0.50, 1.03)	0.92 (0.65, 1.30)	0.80 (0.56, 1.14)	0.465	1.01 (0.89, 1.15)
Model 2	1.00 (ref.)	0.72 (0.50, 1.04)	0.94 (0.67, 1.34)	0.83 (0.58, 1.19)	0.615	1.02 (0.90, 1.17)
**Other fat**						
Median	7.65	11.37	14.91	20.04		
Cases/PYs	73/312,733	67/316,570	57/318,385	55/320,158		
Model 1	1.00 (ref.)	1.01 (0.72, 1.42)	0.96 (0.68, 1.36)	1.00 (0.70, 1.43)	0.933	0.98 (0.86, 1.12)
Model 2	1.00 (ref.)	1.05 (0.75, 1.48)	1.07 (0.75, 1.54)	1.16 (0.80, 1.69)	0.442	1.04 (0.91, 1.19)

SWHS, Shanghai Women’s Health Study; HR, hazard ratio; CI, confidence interval; PYs, person-years; SD, standard deviation; M:S, monounsaturated fat to saturated fat; P:S, polyunsaturated fat to saturated fat; (M + P):S, (monounsaturated and polyunsaturated fat) to saturated fat. ^†^Total and specific dietary fats were adjusted for energy using the nutrient residual model. Model 1 was adjusted by age and calorie intake (quartile). Model 2 adjusted by age (continuous), BMI, education, occupation, income, smoking (yes/no), alcohol consumption (yes/no), tea consumption (yes/no), menopausal status (yes/no), calorie intakes (kcal/day, quartile), physical activity (MET-h/week, quartile), family history of liver cancer (yes/no), personal history of hepatitis (yes/no), cholelithiasis (yes/no), diabetes (yes/no).

Exploratory subgroup analyses were conducted according to BMI and menopausal status in recruited women. After stratifying by both factors, null associations were detected in all fat items, despite the second *vs.* lowest quartile of HR (95% CI) was 0.54 (0.31–0.95) for the P:S ratio in overweight women and the second *vs.* lowest quartile was 0.40 (0.17–0.94) for red meat fat. No significant association was found between the stratified factors (**Table 3**).

**Table 3:** Adjusted HRs for liver cancer incidence stratified by BMI and menopause (SWHS, 1996–2016)

	HR (95% CI)				*P_trend_*	*P_interaction_*
	Q1	Q2	Q3	Q4		
**Total fat**						
BMI (kg/m^2^)						
< 24	1.00 (ref.)	0.91 (0.52, 1.59)	0.92 (0.52, 1.61)	0.93 (0.53, 0.65)	0.837	0.479
≥ 24	1.00 (ref.)	0.73 (0.46, 1.16)	0.75 (0.47, 1.22)	1.27 (0.82, 1.97)	0.426	
Menopause						
No	1.00 (ref.)	0.56 (0.25, 1.26)	0.85 (0.42, 1.74)	0.87 (0.42, 1.78)	0.989	0.485
Yes	1.00 (ref.)	0.90 (0.61, 1.33)	0.82 (0.54, 1.25)	1.24 (0.84, 1.84)	0.458	
**Saturated fat**						
BMI (kg/m^2^)						
< 24	1.00 (ref.)	0.99 (0.55, 1.76)	1.24 (0.71, 2.15)	0.97 (0.54, 1.76)	0.858	0.161
≥ 24	1.00 (ref.)	1.06 (0.69, 1.65)	0.85 (0.52, 1.40)	1.50 (0.96, 2.35)	0.192	
Menopause						
No	1.00 (ref.)	0.73 (0.34, 1.59)	0.87 (0.41, 1.85)	0.99 (0.48, 2.05)	0.866	0.167
Yes	1.00 (ref.)	1.14 (0.78, 1.69)	1.14 (0.76, 1.72)	1.38 (0.92, 2.09)	0.150	
**Monounsaturated fat**						
BMI (kg/m^2^)						
< 24	1.00 (ref.)	0.87 (0.50, 1.51)	0.91 (0.52, 1.59)	0.97 (0.56, 1.70)	0.979	0.648
≥ 24	1.00 (ref.)	1.07 (0.69, 1.65)	0.93 (0.58, 1.49)	1.16 (0.73, 1.85)	0.708	
Menopause						
No	1.00 (ref.)	0.78 (0.37, 1.68)	0.72 (0.33, 1.58)	0.98 (0.48, 1.98)	0.977	0.660
Yes	1.00 (ref.)	1.06 (0.72, 1.55)	1.02 (0.68, 1.53)	1.13 (0.74, 1.71)	0.643	
**Polyunsaturated fat**						
BMI (kg/m^2^)						
< 24	1.00 (ref.)	0.75 (0.41, 1.34)	1.00 (0.58, 1.72)	0.95 (0.54, 1.66)	0.842	0.819
≥ 24	1.00 (ref.)	0.90 (0.57, 1.42)	0.79 (0.49, 1.28)	1.08 (0.69, 1.67)	0.887	
Menopause						
No	1.00 (ref.)	1.09 (0.49, 2.39)	1.16 (0.53, 2.53)	1.35 (0.64, 2.88)	0.417	0.785
Yes	1.00 (ref.)	0.78 (0.52, 1.18)	0.83 (0.55, 1.24)	0.96 (0.65, 1.41)	0.884	
**M:S ratio**						
BMI (kg/m^2^)						
< 24	1.00 (ref.)	0.84 (0.49, 1.44)	0.86 (0.50, 1.48)	0.90 (0.52, 1.56)	0.711	0.582
≥ 24	1.00 (ref.)	0.84 (0.52, 1.35)	0.96 (0.60, 1.52)	0.88 (0.55, 1.40)	0.739	
Menopause						
No	1.00 (ref.)	1.19 (0.57, 2.47)	0.94 (0.43, 2.04)	0.95 (0.44, 2.03)	0.723	0.624
Yes	1.00 (ref.)	0.74 (0.49, 1.12)	0.87 (0.58, 1.29)	0.84 (0.56, 1.26)	0.572	
**P:S ratio**						
BMI (kg/m^2^)						
< 24	1.00 (ref.)	0.99 (0.56, 1.73)	0.98 (0.56, 1.72)	0.97 (0.55, 1.70)	0.906	0.883
≥ 24	1.00 (ref.)	0.54 (0.31, 0.95)	0.97 (0.61, 1.54)	0.92 (0.59, 1.43)	0.669	
Menopause						
No	1.00 (ref.)	0.75 (0.34, 1.64)	1.30 (0.64, 2.63)	1.07 (0.51, 2.26)	0.535	0.783
Yes	1.00 (ref.)	0.72 (0.46, 1.14)	0.88 (0.58, 1.34)	0.89 (0.60, 1.32)	0.869	
**(M + P):S ratio**						
BMI (kg/m^2^)						
< 24	1.00 (ref.)	1.19 (0.69, 2.05)	1.12 (0.64, 1.96)	0.97 (0.54, 1.74)	0.875	0.622
≥ 24	1.00 (ref.)	1.19 (0.71, 1.99)	1.07 (0.64, 1.78)	1.28 (0.79, 2.07)	0.393	
Menopause						
No	1.00 (ref.)	1.36 (0.63, 2.94)	1.41 (0.64, 3.09)	1.39 (0.63, 3.08)	0.438	0.981
Yes	1.00 (ref.)	1.13 (0.73, 1.73)	0.97 (0.63, 1.49)	1.06 (0.70, 1.60)	0.989	
**Food sources**						
**Plant fat**						
BMI (kg/m^2^)						
< 24	1.00 (ref.)	1.02 (0.55, 1.87)	1.35 (0.77, 2.39)	1.26 (0.71, 2.26)	0.283	0.592
≥ 24	1.00 (ref.)	0.83 (0.51, 1.37)	1.08 (0.68, 1.71)	0.93 (0.59, 1.48)	0.963	
Menopause						
No	1.00 (ref.)	0.47 (0.20, 1.11)	0.97 (0.48, 1.93)	1.01 (0.50, 2.03)	0.613	0.726
Yes	1.00 (ref.)	1.08 (0.69, 1.67)	1.26 (0.83, 1.92)	1.07 (0.70, 1.64)	0.623	
**Red meat fat**						
BMI (kg/m^2^)						
< 24	1.00 (ref.)	0.58 (0.32, 1.04)	0.93 (0.54, 1.59)	0.82 (0.47, 1.42)	0.898	0.671
≥ 24	1.00 (ref.)	0.82 (0.52, 1.30)	0.96 (0.61, 1.51)	0.83 (0.52, 1.34)	0.616	
Menopause						
No	1.00 (ref.)	0.40 (0.17, 0.94)	0.73 (0.35, 1.51)	0.78 (0.39, 1.54)	0.852	0.706
Yes	1.00 (ref.)	0.84 (0.56, 1.26)	1.04 (0.70, 1.54)	0.83 (0.54, 1.28)	0.665	
**Other fat**						
BMI (kg/m^2^)						
< 24	1.00 (ref.)	0.87 (0.49, 1.54)	0.87 (0.49, 1.57)	1.10 (0.63, 1.94)	0.688	0.517
≥ 24	1.00 (ref.)	1.15 (0.75, 1.75)	1.19 (0.75, 1.88)	1.11 (0.67, 1.86)	0.575	
Menopause						
No	1.00 (ref.)	1.42 (0.62, 3.23)	1.46 (0.64, 3.33)	1.73 (0.77, 3.91)	0.208	0.688
Yes	1.00 (ref.)	1.01 (0.69, 1.47)	1.03 (0.69, 1.55)	1.09 (0.71, 1.69)	0.692	

SWHS, Shanghai Women’s Health Study; HR, hazard ratio; CI, confidence interval; BMI, body mass index. Total and specific dietary fats were adjusted for energy using the nutrient residual model. All were adjusted by age (continuous), BMI, education, occupation, income, smoking (yes/no), alcohol consumption(yes/no), tea consumption(yes/no), menopausal status (yes/no), calorie intakes (kcal/day, quartile), physical activity (MET-h/week, quartile), family history of liver cancer (yes/no), personal history of hepatitis (yes/no), cholelithiasis (yes/no), T2DM (yes/no). Of note, the variables examined in this table were not adjusted.

Finally, 3 strategies of sensitivity analyses were conducted. We found that after excluding participants who had less than 2 years of follow-up, similar results were observed except for the P:S ratio and red meat fat. The second *vs.* lowest quartile of HRs (95% CIs) were 0.60 (0.39–0.92) for the P:S ratio and 0.65 (0.44–0.96) for the red meat fat, with corresponding *P*-trend values of 0.827 and 0.732, respectively. Null associations were found after excluding participants with T2DM at baseline recruitment. After applying the density-energy method to adjust for dietary fat intake, we found null associations for all dietary fats except for the (M + P):S ratio; the second *vs.* lowest quartile of HR (95% CI) was 1.62 (1.10–2.37) with a *P*-trend value of 0.436 (**Table 4**).

**Table 4:** Sensitivity analyses: HRs and 95% CIs for liver cancer incidence (SWHS, 1996–2016)

	HR (95% CI)				*P_trend_*
	Q1	Q2	Q3	Q4	
**Excluding the first 2 years of follow-up^†^ (222 cases in total)**					
Total fat	1.00 (ref.)	0.81 (0.56, 1.17)	0.78 (0.53, 1.15)	1.04 (0.72, 1.50)	0.984
Saturated fat	1.00 (ref.)	1.01 (0.70, 1.46)	1.07 (0.74, 1.57)	1.15 (0.78, 1.69)	0.457
Monounsaturated fat	1.00 (ref.)	1.01 (0.70, 1.45)	0.87 (0.59, 1.28)	1.07 (0.73, 1.56)	0.969
Polyunsaturated fat	1.00 (ref.)	0.84 (0.57, 1.22)	0.88 (0.60, 1.28)	0.97 (0.67, 1.40)	0.916
M:S ratio	1.00 (ref.)	0.87 (0.60, 1.28)	0.94 (0.64, 1.37)	0.94 (0.64, 1.37)	0.849
P:S ratio	1.00 (ref.)	0.60 (0.39, 0.92)	0.88 (0.60, 1.28)	0.92 (0.64, 1.32)	0.827
(M + P):S ratio	1.00 (ref.)	1.07 (0.71, 1.60)	1.03 (0.69, 1.54)	1.20 (0.82, 1.76)	0.379
Food sources					
Plant fat	1.00 (ref.)	0.87 (0.57, 1.31)	1.24 (0.85, 1.81)	1.02 (0.69, 1.50)	0.500
Red meat fat	1.00 (ref.)	0.65 (0.44, 0.96)	0.87 (0.60, 1.26)	0.85 (0.59, 1.24)	0.723
Other fat	1.00 (ref.)	1.03 (0.72, 1.47)	1.01 (0.69, 1.48)	1.04 (0.70, 1.56)	0.873
**Excluding participants with diabetes at recruitment^†^ (227 cases in total)**					
Total fat	1.00 (ref.)	0.76 (0.53, 1.10)	0.75 (0.52, 1.10)	1.05 (0.73, 1.51)	0.970
Saturated fat	1.00 (ref.)	0.95 (0.66, 1.37)	0.98 (0.68, 1.43)	1.18 (0.81, 1.72)	0.430
Monounsaturated fat	1.00 (ref.)	0.96 (0.67, 1.36)	0.93 (0.64, 1.35)	0.98 (0.67, 1.44)	0.868
Polyunsaturated fat	1.00 (ref.)	0.86 (0.59, 1.25)	0.88 (0.61, 1.28)	1.07 (0.75, 1.54)	0.721
M:S ratio	1.00 (ref.)	0.76 (0.52, 1.11)	0.84 (0.58, 1.21)	0.89 (0.62, 1.29)	0.702
P:S ratio	1.00 (ref.)	0.70 (0.46, 1.07)	1.00 (0.69, 1.46)	0.99 (0.68, 1.43)	0.588
(M + P):S ratio	1.00 (ref.)	1.06 (0.72, 1.58)	0.99 (0.67, 1.48)	1.18 (0.81, 1.71)	0.465
Food sources					
Plant fat	1.00 (ref.)	0.99 (0.66, 1.46)	1.27 (0.87, 1.84)	1.10 (0.75, 1.61)	0.385
Red meat fat	1.00 (ref.)	0.75 (0.51, 1.09)	0.90 (0.63, 1.31)	0.77 (0.53, 1.14)	0.352
Other fat	1.00 (ref.)	1.00 (0.70, 1.43)	1.05 (0.72, 1.53)	1.13 (0.76, 1.68)	0.537
**Using the energy density method to re-analyze the full cohort**					
Total fat	1.00 (ref.)	0.97 (0.69, 1.37)	1.00 (0.69, 1.43)	1.23 (0.86, 1.76)	0.295
Saturated fat	1.00 (ref.)	0.92 (0.65, 1.30)	0.95 (0.66, 1.37)	1.28 (0.90, 1.81)	0.209
Monounsaturated fat	1.00 (ref.)	0.86 (0.61, 1.21)	0.96 (0.68, 1.37)	1.10 (0.77, 1.57)	0.561
Polyunsaturated fat	1.00 (ref.)	1.04 (0.73, 1.49)	1.07 (0.75, 1.53)	1.17 (0.82, 1.67)	0.379
M:S ratio	1.00 (ref.)	0.91 (0.64, 1.28)	0.91 (0.64, 1.29)	0.91 (0.64, 1.29)	0.613
P:S ratio	1.00 (ref.)	0.80 (0.54, 1.18)	1.01 (0.71, 1.44)	1.00 (0.70, 1.41)	0.681
(M + P):S ratio	1.00 (ref.)	1.62 (1.10, 2.37)	1.35 (0.91, 1.99)	1.31 (0.89, 1.92)	0.436
Food sources					
Plant fat	1.00 (ref.)	0.89 (0.61, 1.28)	1.10 (0.78, 1.56)	0.97 (0.68, 1.38)	0.858
Red meat fat	1.00 (ref.)	0.75 (0.53, 1.06)	0.93 (0.67, 1.30)	0.83 (0.58, 1.19)	0.488
Other fat	1.00 (ref.)	0.94 (0.67, 1.33)	1.01 (0.70, 1.44)	1.06 (0.73, 1.53)	0.736

SWHS, Shanghai Women’s Health Study; HR, hazard ratio; CI, confidence interval; M:S, monounsaturated fat to saturated fat; P:S, polyunsaturated fat to saturated fat; (M + P):S, (monounsaturated and polyunsaturated fat) to saturated fat. ^†^Total and specific dietary fats were adjusted for energy using the nutrient residual model. All adjusted by age (continuous), BMI, education, occupation, income, smoking (yes/no), alcohol consumption (yes/no), tea consumption (yes/no), menopausal status (yes/no), calorie intakes (kcal/day, quartile), physical activity (MET-h/week, quartile), family history of liver cancer (yes/no), personal history of hepatitis (yes/no), cholelithiasis (yes/no), diabetes (yes/no).

## Discussion

In this prospective population-based cohort study, we assessed the associations between dietary fats and liver cancer incidence in Chinese women. Among 72,704 participants with 1,267,845 person-years of follow-up, 252 females were newly diagnosed with liver cancer. No significant association between dietary fats and liver cancer risks was detected, and even in the stratification and sensitivity analyses, similar results were observed.

To date, studies on the associations between dietary fat and liver cancer risks are very limited, particularly in women. Similar to the results reported in the US cohort study^14^ that performed a stratification analysis by sex, null associations were found between total fat and HCC risk, with decreased risks (ranging from 0.82–0.73) across quartiles in women in the NHS, whereas the risks (ranging from 0.97–1.23) were higher in our study. Taking the risk of 1-SD increment into consideration, the HR (95% CI) was 0.92 (0.72–1.19) for total fat in the NHS^14^ but 1.03 (0.90–1.17) in our SWHS. Discrepancies in risks were also observed when comparing the NHS^14^ and SWHS; the highest *vs*. lowest quartile of HRs were 0.73 *vs*. 1.11 for total fat, 0.56 *vs.* 1.25 for saturated fat, 0.71 *vs.* 1.08 for MUFA, and 0.59 *vs*. 1.02 for PUFA (all *P*-trend values were > 0.05). In the European Prospective Investigation into Cancer and Nutrition cohort study (EPIC), the highest *vs.* lowest quartile of HRs (95% CI) were 0.74, 1.19, 0.53, and 0.86 for total fat, saturated fat, MUFA, and PUFA (all *P*-trend values were > 0.05), respectively^29^. In the National Institute of Health-AARP (NIH-AARP) Diet and Health Study, the HR of 5% increase in energy was statistically significant with 1.10 in total fat, and 1.38 for saturated fat; and not significant with 1.18 for MUFA and 1.06 for PUFA in the whole population^30^. In the Singapore Chinese Health cohort, the highest *vs.* lowest quartiles of HRs were 1.26 for total fat, 1.40 for saturated fat, and 0.90 for MUFA, with all HRs being insignificant^31^. All the above cohort studies were conducted for both sexes and not specifically in women. The possible explanations for these combined analyses might be related to the similar risk factor exposures between men and women, as well as the limited number of incident cases in women.

Considering the disparities of results in women from the NHS and our SWHS, the causes may be attributed to food sources of dietary fat. In the NHS, vegetable fat was the second most common food source and had the potential ability to decrease HCC risk^14^; whereas, it was far less in our SWHS (data not shown). In addition, the discrepancies between our study results and the EPIC and NIH-AARP may be attributed to sex differences. As indicated by the Shanghai Men’s Health Study^32^, dietary fat was associated with a higher risk of liver cancer in Chinese men. Furthermore, this inconsistency may partly be explained by different dietary habits. As shown in a recent review^33^, the main food sources of MUFA were meat and meat products in the NIH-AARP, and olive oil and nuts (~ 20%) in the EPIC study, and fats from olive oil and nuts were associated with lower risks of HCC. Comparing our results with those from other cohort studies, the Singapore Chinese Health Study reported the most similar conclusions, except for the HR for MUFA. Because all analyses from the EPIC, NIH-AARP, and Singapore Chinese Health Study were performed using both sexes, it might also imply that sex and ethnicity had some effects on the associations between dietary fat and liver carcinogenesis.

Taking into account fat subtype ratios, the null significance was detected in associations between M:S, P:S, and (M + P):S ratios and liver cancer risk in the NHS or our SWHS. After stratifying using the BMI, a null association was observed in our SWHS, which was similar to the result that was obtained in the whole analyses of all participants from the NHS (women cohort) and the HPFS (men cohort)^14^. As presented in the clinical trial of the Dietary Intervention Study in Children^34^, the P:S ratio was inversely associated with breast density, which could serve as a strong risk factor for breast cancer in women. The *n*-3 and *n*-6 are the main types of PUFAs. A report from epidemiological evidence and an animal model suggested that increasing *n*-3 PUFAs and decreasing *n*-6 PUFAs and saturated fat intakes had potential effects on colon cancer treatment^35^. All the above studies showed the importance of fat composition ratios in cancer incidence and prevention. However, it also implied that not all PUFAs were good for health, and further detailed analyses should be conducted.

Dietary fat digestion starts in the oral cavity by lingual lipase exposure. The digestion continues in the stomach *via* the lingual and gastric enzymes, and after crude emulsion with the contribution of gastric peristalsis, fine lipid droplets are formed, then they enter into the duodenum, undergo the effects of bile acid and pancreatic enzymes, and then the chemical and physical structures are changed to help digestion^36^. Shen et al.^37^ studied the influences of dietary fat composition (high fat *vs.* low fat) on intestinal microbiota and showed that the subsequent fermentation products affected liver function. In C57BL/6J mice, a high-fat diet could induce the progression of non-alcoholic fatty liver disease (NAFLD) to non-alcoholic steatohepatitis and subsequently to HCC^38^. The *n*-3 and *n*-6 PUFAs are the main types of PUFA, and different effects for liver diseases have been previously reported. As reported by the NHS and HPFS studies, high dietary intake of PUFAs was associated with a reduced risk of HCC in U.S. adults^14^. Another report suggested that the *n*-3 PUFAs seemed to reduce HCC risk, even among subjects with HBV/HCV infection^39^. However, *n*-6 PUFAs promoted inflammatory responses, leading to the increased production of inflammatory cytokines by liver Kupffer cells and the activation of NF-κB, thus aggravating liver inflammation and fibrosis^40^. Collectively, specific fat compositions may have different effects on hepatocellular carcinogenesis, which should be explored in-depth in future studies.

### Advantages and limitations

This is the first cohort study that evaluated the associations between dietary fat and liver cancer risk in Chinese women. The advantages of this study were related to the large-scale, population-based, and prospective cohort study design. This study represented a prospective cohort study with the largest number of liver cancer incident cases in women that had currently been reported. In addition, multiple confounding factors were included in this study, such as personal medical histories of hepatitis, cholelithiasis, T2DM, and family history of liver cancer, which could be potential causes of liver cancer. Meanwhile, this study presented some additional limitations as well. First, no dietary alterations during follow-up were considered, and baseline FFQ surveys were adopted for the analyses. In addition, the measurement bias of dietary consumption may have led to biased results. Furthermore, as the main source of dietary fat, oil consumption was not exactly calculated. For the shortage of early FFQ design, monthly family but not daily individual oil consumption data were collected. Considering that the oil fat data were crude and not as accurate as the dietary fat data, we included the combined results of dietary fat and oil fat in the supplementary material to support our conclusions. Another limitation of our study was the lack of recording HBV or HCV infection, which were the major causes of HCC. However, as one of the main outcomes of HBV or HCV infection, the medical history of chronic hepatitis has been calibrated in the multivariate model, which could, to some extent, reduce this bias.

## Conclusions

In summary, our results showed no significant association between total dietary fat, specific fat subtypes, and food sources, and liver cancer risk in Chinese women. Due to the limited number of related studies, more epidemiological evidence should be obtained to further investigate the role of dietary fat intake in liver carcinogenesis in women.
