# Supplementary material for: Dietary fat intake and liver cancer risk: A prospective cohort study in Chinese women
Source: Cancer Biol Med. 2021 Sep 21;19(3):370–83. doi: 10.20892/j.issn.2095-3941.2020.0633 (PMC8958890; doi:10.20892/j.issn.2095-3941.2020.0633)
Supplement: Supplementary file 1 [file cbm-19-370-s001.pdf]

Supplementary material

Table S1 Adjusted HRs and 95% CIs of liver cancer by quartiles of energy-adjusted† intakes of dietary fat and oil fat (SWHS, 1996–2016)

	HR (95% CI)				P_{trend}	HR (95% CI) for 1-SD increment
	Q1	Q2	Q3	Q4		
**Total fat**						
Median	50.18	60.02	67.99	80.00		
Cases/PYs	67/313,543	62/312,661	59/311,033	63/308,342		
Model 1	1.00 (ref.)	0.97 (0.68, 1.37)	0.93 (0.66, 1.33)	1.06 (0.75, 1.50)	0.804	0.99 (0.87, 1.12)
Model 2	1.00 (ref.)	0.99 (0.70, 1.40)	0.93 (0.65, 1.32)	1.03 (0.73, 1.46)	0.981	0.98 (0.86, 1.11)
**Saturated fat**						
Median	10.49	12.97	14.92	17.81		
Cases/PYs	72/310,729	62/312,193	52/311,697	65/310,960		
Model 1	1.00 (ref.)	0.92 (0.65, 1.30)	0.82 (0.57, 1.17)	1.11 (0.79, 1.55)	0.744	1.02 (0.90, 1.15)
Model 2	1.00 (ref.)	0.95 (0.67, 1.34)	0.85 (0.59, 1.23)	1.16 (0.82, 1.64)	0.572	1.03 (0.91, 1.18)
**Monounsaturated fat**						
Median	16.07	19.82	22.71	27.21		
Cases/PYs	73/311,603	62/312,149	59/310,987	57/310,839		
Model 1	1.00 (ref.)	0.89 (0.63, 1.25)	0.90 (0.64, 1.28)	0.96 (0.68, 1.37)	0.830	1.02 (0.90, 1.16)
Model 2	1.00 (ref.)	0.90 (0.63, 1.26)	0.92 (0.65, 1.31)	0.98 (0.69, 1.39)	0.926	1.03 (0.91, 1.17)
**Polyunsaturated fat**						
Median	20.73	25.64	29.98	36.93		
Cases/PYs	70/315,963	50/313,152	70/309,261	61/307,202		
Model 1	1.00 (ref.)	0.71 (0.49, 1.02)	0.95 (0.68, 1.32)	0.84 (0.60, 1.19)	0.653	0.99 (0.87, 1.12)
Model 2	1.00 (ref.)	0.69 (0.48, 0.99)	0.90 (0.64, 1.26)	0.76 (0.53, 1.08)	0.299	0.95 (0.84, 1.07)
**M:S ratio**						
Median	1.37	1.49	1.57	1.69		
Cases/PYs	69/313,796	57/311,068	67/309,243	58/311,471		
Model 1	1.00 (ref.)	0.80 (0.57, 1.14)	0.89 (0.63, 1.25)	0.85 (0.60, 1.21)	0.494	0.99 (0.89, 1.11)
Model 2	1.00 (ref.)	0.75 (0.53, 1.07)	0.83 (0.58, 1.17)	0.81 (0.57, 1.15)	0.342	0.98 (0.89, 1.08)
**P:S ratio**						
Median	1.53	1.89	2.19	2.62		
Cases/PYs	53/316,493	61/313,139	69/310,114	68/305,832		
Model 1	1.00	1.06 (0.73, 1.54)	1.11 (0.77, 1.59)	0.99 (0.69, 1.42)	0.976	0.99 (0.91, 1.08)
Model 2	1.00 (ref.)	1.01 (0.70, 1.47)	0.99 (0.68, 1.43)	0.84 (0.57, 1.22)	0.331	0.98 (0.93, 1.03)

Table S1 Continued

	HR (95% CI)				P_{trend}	HR (95% CI) for 1-SD increment
	Q1	Q2	Q3	Q4		
**(M + P):S ratio**						
Median	2.99	3.41	3.77	4.23		
Cases/PYs	58/316,018	67/313,397	56/310,123	70/306,040		
Model 1	1.00 (ref.)	1.09 (0.76, 1.55)	0.84 (0.58, 1.21)	0.96 (0.68, 1.37)	0.513	0.99 (0.92, 1.07)
Model 2	1.00 (ref.)	1.02 (0.71, 1.45)	0.74 (0.51, 1.09)	0.80 (0.55, 1.16)	0.106	0.98 (0.94, 1.03)
**Food source**						
**Plant fat**						
Median	2.66	4.44	6.12	9.17		
Cases/PYs	55/313,783	56/312,123	73/310,604	67/309,068		
Model 1	1.00 (ref.)	0.96 (0.66, 1.40)	1.20 (0.84, 1.71)	1.06 (0.74, 1.51)	0.500	1.00 (0.89, 1.14)
Model 2	1.00 (ref.)	0.94 (0.64, 1.38)	1.17 (0.82, 1.68)	1.01 (0.70, 1.45)	0.678	0.98 (0.87, 1.11)
**Red meat fat**						
Median	2.81	6.43	9.50	15.32		
Cases/PYs	72/309,775	58/310,331	63/311,706	58/313,766		
Model 1	1.00 (ref.)	0.77 (0.54, 1.11)	0.87 (0.61, 1.24)	0.90 (0.64, 1.28)	0.730	1.02 (0.90, 1.16)
Model 2	1.00 (ref.)	0.80 (0.56, 1.15)	0.91 (0.64, 1.30)	0.96 (0.68, 1.37)	0.999	1.04 (0.91, 1.18)
**Oil fat**						
Median	22.93	31.13	38.72	51.27		
Cases/PYs	70/316,689	53/312,772	64/309,024	64/307,093		
Model 1	1.00 (ref.)	0.75 (0.52, 1.08)	0.85 (0.60, 1.19)	0.89 (0.63, 1.25)	0.626	0.98 (0.87, 1.11)
Model 2	1.00 (ref.)	0.72 (0.50, 1.04)	0.78 (0.55, 1.09)	0.79 (0.55, 1.11)	0.244	0.94 (0.82, 1.06)

This analysis was performed in 71,408 participants (excluding 1,296 participants who had missing or extreme oil data (keep participants with 1%–99% of oil fat). SWHS, Shanghai Women's Health Study; HR, hazard ratio; CI, confidence interval; PYs, person-years; SD, standard deviation; M:S, monounsaturated fat to saturated fat; P:S, polyunsaturated fat to saturated fat; (M + P):S, (monounsaturated and polyunsaturated fat) to saturated fat. †Total and specific dietary fats were adjusted for energy using the nutrient residual model. Model 1 was adjusted by age and calorie intake (quartile). Model 2 adjusted by age (continuous), BMI, education, occupation, income, smoking (yes/no), alcohol consumption (yes/no), tea consumption (yes/no), menopausal status (yes/no), calorie intakes (kcal/day, quartile), physical activity (MET, hours/week, quartile), family history of liver cancer (yes/no), personal history of hepatitis (yes/no), cholelithiasis (yes/no), diabetes (yes/no).
